# Relational autonomy in breast diseases care: a qualitative study of contextual and social conditions of patients’ capacity for decision-making

**DOI:** 10.1186/s12913-018-3622-8

**Published:** 2018-10-25

**Authors:** Patti Shih, Frances Rapport, Anne Hogden, Mia Bierbaum, Jeremy Hsu, John Boyages, Jeffrey Braithwaite

**Affiliations:** 1 Centre for Healthcare Resilience and Implementation Science, Australian Institute of Health Innovation, Faculty of Health and Medical Sciences, Macquarie University, Sydney, NSW Australia; 2 Department of Clinical Medicine, Faculty of Health and Medical Sciences, Macquarie University, Sydney, NSW Australia

**Keywords:** Relational autonomy, Patient autonomy, Treatment decision-making, Person-centred care, Breast disease care, Breast cancer care

## Abstract

**Background:**

A relational approach to autonomy refers to the way in which social conditions and relationships shape a person’s self-identity and capacity in decision-making. This article provides an empirical account of how treatment choices for women undergoing breast diseases care are fostered within the dynamics of their relationships with clinicians, family members, and other aspects of their social environment.

**Methods:**

This qualitative study recruited ten women undergoing treatment at a breast programme, and eight clinicians supporting their care, in a private teaching hospital in New South Wales, Australia. Fourteen patient-clinician consultation observations and 17 semi-structured interviews were conducted. Schema analysis of interview transcripts were undertaken by a team of researchers and corroborated by observational fieldnotes.

**Results:**

Relational identities of patients influenced the rationale for treatment decision-making. Patients drew on supportive resources from family and medical advice from clinicians to progress with treatment goals. While clinicians held much social power over patients as the medical experts, patients highlighted the need for clinicians to earn their trust through demonstrated professionalism. Information exchange created a communicative space for clinicians and patients to negotiate shared values, promoting greater patient ownership of treatment decisions. As treatment progressed, patients’ personal experiences of illness and treatment became a source of self-reflection, with a transformative impact on self-confidence and assertiveness.

**Conclusion:**

Patients’ confidence and self-trust can be fostered by opportunities for communicative engagement and self-reflection over the course of treatment in breast disease, and better integration of their self-identity and social values in treatment decisions.

**Electronic supplementary material:**

The online version of this article (10.1186/s12913-018-3622-8) contains supplementary material, which is available to authorized users.

## Background

Patient autonomy in the context of healthcare refers to the ability of individuals to independently determine the path of their own treatment [1, 2]. It is a fundamental principle of person-centred care, which promotes the reflection of patients’ personal values and preferences in treatment decisions. The promotion of personal autonomy has focused on ensuring that patients are well informed of the full range of options available to them, including the benefits and consequences of treatment choices. Removing barriers against patients’ ability to make independent decisions, such as the potentially paternalistic and coercive pressures from clinicians and close family members, are also emphasised [3–5].

The concept of relational autonomy arose from feminist critiques of an over-individualised understanding of personal autonomy [6–9]. Individuals are socially embedded, thus their identities are formed within the context of social relationships and shaped by complex intersecting social determinants, such as gender, class and ethnicity [6]. A narrow focus on individualist notions of self-determination and independence can downplay the social conditions that structure decisions made in healthcare settings [7]. These include socially constructed power relationships, such as gender norms that shape attitudes of body image and health, socio-economic conditions that alter the access to healthcare knowledge and services, and the lack of social power that depletes self-trust and confidence to question and challenge the opinions of treating doctors [7, 10].

At the same time, not all social conditions that underscore autonomy are limiting, but rather, can foster a person’s capacity to be autonomous. A ‘capabilities approach’ [11, 12] suggests that resources and opportunities in a person’s relational world can provide a social environment which strengthens self-identity, self-determination and autonomy. Clinicians, close family members and carers are ‘important social others’ [12] in a patient’s treatment and care. These social relationships can develop opportunities for communication and social interactions that fosters patients’ active engagement and dialogue in treatment decision-making [13–15].

Given the emphasis on social context and the conditional environment in which individuals exist, relational autonomy is best illustrated by studying the empirical setting in which it is practised. The treatment of breast diseases for women is an appropriate context for examining relational autonomy in decision-making. Breast disease care, including for breast cancer and benign tumours, is complicated by the availability of multiple treatment options and a potentially lengthy care continuum [16–18]. Previous studies have noted the clinical as well as social and personal issues that face women in deciding to undergo the range and succession of adjuvant therapies such as different types of surgery, chemotherapy, radiation therapy, and hormone treatments. With each treatment stage and the emerging medical options, there is often an increasing uncertainty about patient and clinical outcomes, and the marginal benefits of some treatments may come with potential risks of complications and side-effects [17, 19, 20]. The gendered concerns about body image and sexuality, and the anxieties around the well-being for loved ones while patients’ lives are interrupted by illness, are often enmeshed in breast disease treatment decision-making [17–19, 21].

In view of this, the need to ensure patients’ values and preferences are met in treatment decision-making is particularly pronounced [19, 22]. This study provides an account of women’s treatment decision-making in breast disease care as contextualised by the dynamics in their key relationships with family and clinicians, and their wider social environment.

## Methods

This qualitative study took place at a breast program in a private, not-for-profit teaching hospital, located on a university campus in New South Wales, Australia. The study was approved by the relevant human research ethics committee. The programme treats patients with invasive cancer, ductal carcinoma in situ (DCIS), and benign disease. Patient recruitment was conducted according to a timeframe sampling approach, which allocates a specifically chosen window of time during which recruitment takes place. It is a method of choice to avoid researcher or clinician coercion in sampling decisions, and offers opportunities for maximum variation among participants. The first ten patients undergoing treatment at the study site that consented to take part between August 2016 and January 2017 were sampled. Inclusion criteria stipulated that patients needed to be female, competent English speakers, over the age of 18, and deemed physically and mentally capable of participating by clinicians. Eight clinicians within the programme who played a role in caring for one or more of the patients were also recruited. Although the focus of this article is on patients diagnosed with cancer and their treatment decision-making, cases of both benign disease and DCIS, were included to best demonstrate the importance of relational factors that influence personal capacity for decision-making. The demographic profile of patients is provided in Table 1. All quotes attributed to participants in the results section are de-identified by the assignment of pseudonyms in italics.

**Table 1:** Patient participants’ demographic profiles

Participant Pseudonym	Age	Stage/Diagnosis	Treatment Plan and Sequence	Marital status and children	Employment status
*Annette*	56	Stage 2 cancer	1. Chemotherapy 2. Lumpectomy 3. Radiation	Married; 3 adult children	Full-time employed
*Bonny*	52	Stage 0 Ductal Carcinoma in situ (DCIS)	1. Lumpectomy 2. Radiation	Married; No children	Full-time employed
*Caitlyn*	46	Stage 3 cancer	1. Chemotherapy 2. Lumpectomy 3. Radiation	Married; 3 dependent children	Part-time employed
*Diedre*	55	Stage 2 cancer	1. Lumpectomy 2. Radiation	Divorced;1 dependent child	Self-employed
*Ella*	35	Stage 2 cancer	1. Chemotherapy 2. Lumpectomy 3. Radiation	Married; 3 dependent children	Self-employed
*Frederika*	64	Stage 1 cancer	1. Lumpectomy 2. Radiation	Separated; 2 adult children	Full-time employed
*Gladys*	68	Stage 1 cancer	1. Mastectomy 2. Chemotherapy 3. Radiation	Married; 2 adult children	Retired
*Hillary*	72	Stage 2 cancer	First cancer (right breast) 1. Chemotherapy 2. Lumpectomy 3. Radiation Second cancer (left breast) 1. Mastectomy	Married; 3 adult children	Retired
*Ingrid*	28	Fibroadenoma (benign breast lump)	1. Lumpectomy	Married; No children	Part-time employed
*Jane*	34	Stage 2 cancer	1. Lumpectomy 2. Radiation	Married; No children	Full-time employed

Fourteen clinical consultations were observed to examine patient-professional communication, including the timing of discussions, the language used during consultations, and the intentions expressed. Observation fieldnotes were taken in order to support a fuller record of the observational event. Seventeen semi-structured interviews were conducted with patients and clinicians separately following observed consultations, using an interview guide designed by the research team (see Additional file 1). They lasted approximately 45 min, and were audio-recorded and notated, before being transcribed verbatim.

A schema analysis of transcribed data was conducted. Schema analysis is a qualitative data analysis technique that uses a summative approach to clarify textual data, ensuring the essential elements of data are captured in one, free flowing, narrative text, before being refined into a representation of the core concepts inherent in the data [23, 24]. Firstly, four researchers each provided a succinct overview of the complete content of each transcript, concentrating on the meaning of the text from the perspective of the research participants and indicating the text’s essential meaning for them. Secondly, the four researchers worked collaboratively to succinctly summarise and analyse together a sub-set of the dataset, led by a senior academic experienced in schema analysis. Team members compared and contrasted their individually crafted schema, to arrive at a consensus of opinion on the most essential elements within each transcribed text. Observational notes and interview transcripts were considered corroboratively, with notations from observed events enhancing the accuracy and add validity to the data analysis process.

Findings presented in this article concentrate on the patient’s perspective. Clinicians’ perspectives and observational data, when applicable, are introduced to contextualise the patients’ views.

## Results

The study results are assembled into five main themes: relational identities and relational resources, clinician-patient relationships, negotiating shared values, lack of clarifying values, and gaining experience and self-reflection over-time.

### Relational identities and relational resources

‘It was a matter of life and death. I want to live’ (*Hillary*). The urgency to undergo treatment after an unexpected cancer diagnosis is overwhelmingly understood by women as a matter of personal survival. Even so, the implications of survival were also defined in relation to women’s socially and personally valued identities as mothers, grandmothers, wives and partners in a family unit: ‘I’ve got three beautiful girls … I just want to survive’ (*Caitlyn*), and: ‘I want to be here to see my granddaughter get married’ (*Annette*).

On the other hand, patients highlighted how their loved ones reciprocated support in a time of vulnerability and personal crisis. In recognition of their diminished capacity for making reasoned decisions at a time of emotional upheaval as they learn about a cancer diagnosis, patients drew emotional and logistical support from close family members to help them make progress in treatment. *Annette* ‘didn’t want to see anyone or do anything’ in the days following diagnosis, and it was her adult son who contacted the oncologist on her behalf to initiate the first treatment assessment. *Ella* relied on her husband and sister-in-law to take notes during her oncology consultations as she was: ‘too messed up to take anything in’, while *Caitlyn* delegated to her husband to synthesise treatment information for her when she felt that ‘too much detail was too scary’ at a time of heightened anxiety. As some patients acknowledged that their own sense of judgement was compromised by the trauma of an unexpected cancer diagnosis, family support enabled them to keep focussed on the need to begin treatment and: ‘think clearly’ *(Ella)*.

Apart from close family, the workplace also became an important source of support. Other than two retirees, all patients were engaged in a professional career that they valued as part of their self-identity, and noted their appreciation for colleagues and employees who were willing to provide them with extended sick leave and emotional encouragement while cancer treatment interrupted their careers and lives.

Notably, the stage of breast disease at the time of diagnosis was not found to be the determining factor of a patient’s feeling of shock and perceived impairment in decision-making, nor their level of need in reliance and support from family and the wider community. Rather, each patient’s personal situation and quality of inter-personal relationships determined this dynamic.

### Clinician-patient relationships: a communicative space for building trust

Patients’ relationships with their clinicians is a crucial partnership, and evolved over the course of clinical treatment.

As trained professionals with specialised medical knowledge and expertise in breast disease, clinicians according to many accounts occupy a socially privileged position of power in relation to their patients [7, 25]. This power relationship was particularly evident at the start of the treatment, as patients were new to understanding their diagnosed disease and looked for expert direction and advice. However, they must also facilitate the development of trust through their interaction with patients.

In the observed clinical consultations, clinicians routinely provided patients with technical information in relation to their disease and diagnosis, such as details of tumour type, tumour size, position, and stage of invasiveness, and the risks and benefits of the treatment options available. Yet when interviewed, patients rarely mentioned the technical details of prognosis and treatment conveyed by clinicians. Instead, many noted the trust and confidence they bestowed upon clinicians to lead the treatment decisions, and highlighted professional thoroughness and scientific knowledge demonstrated by clinicians in the way they provided the rationale for the treatment plans: ‘[Clinician] knew what he was talking about. He has written a book, after all’ *(Bonny).* The perception of professionalism and competence demonstrated by clinicians in this respect, gave patients a sense of relief that their health and survival was in the hands of: ‘the best team’ *(Ella).*

What patients most valued in these clinical consultations, rather than the content of the medical information discussed, was the chance to establish ‘trust and rapport’ with clinicians, especially at the start of a life-altering treatment journey. A chance to engage in what was seemingly “small talk” with patients allowed clinicians to understand a patient’s personal circumstances, lifestyle and leisure activities, professional work demands and family situation. This information was in fact crucial in helping clinicians formulate and tailor treatment advice to the patients’ personal circumstances at this particular time in their lives. For example, the readiness and appropriateness for intensive and lengthy radiation therapy or breast-preserving surgery may not be the same for a younger patient in her 30s as it is for and older patient, or a patient with an active lifestyle, professional work load, or worried about fertility or caring for young children. In turn, the early establishment of personal trust and comfort with the medical team through interpersonal rapport was crucial in securing patients’ commitment to progress with treatment.

Therefore, rather than questioning the legitimacy of the treatment plans on offer or prognosis provided, patients were more interested in ensuring that they played their role in optimising treatment success. A large proportion of questions posed by patients centred on the logistics of treatment, such as the day-to-day practicalities of hospital parking during regular treatment visits, scheduling treatment visits to suit work arrangements, and how one can manage side-effects of radiation or chemotherapy such as skin-burning, hair-loss and nail-damage: ‘As long as they tell me what to do - what lotions to put on it, drink water. As long as they tell me what to do, I will do it.’ *(Ella).* For patients, this was their way of reciprocating trust, by reassuring clinicians that they were also willing and committed to the treatment process.

An individualistic approach to patient autonomy takes a functional and transactional view of information provision, as a way to obtain patients’ informed choice based on the rational deliberation of the cost and benefits of treatment options [8, 10]. Therefore, the preferences of clinicians were seen as potentially interfering with patient’s autonomy. However, patients in this study did not suggest that their adherence to clinicians’ guidance was coercive, but rather, conditional, once they felt clinicians had demonstrated professional competence and deserved their trust. In fact, when presiding clinicians failed to quickly establish trust and confidence, patients became wary. One patient had deliberately sought the second opinion of her current treating clinician, when her first oncologist from another hospital failed to communicate well with her, nor provided her with a sense of confidence with their skills and knowledge. Rather than a passive recipient of clinicians’ directives, this patient showed that she had a degree of power in ascertaining the appropriate medical team to steer her treatment, despite admitting that by changing doctors and hospitals, she had ‘upset the apple cart’.

### Negotiating shared values

Clinicians gained patients’ trust not only by demonstrating their professional expertise and grasp of medical knowledge. Their ability to facilitate a communicative space opens an opportunity for dialogue between clinicians and patients. This allowed clinicians and patients to acquaint with each other’s values and points of view. For example, clinicians detailed the treatment approach they advised, based on their medical expertise and knowledge, while giving patients an opportunity to express their values in relation to their advice, and vice versa. The opportunity for patients to ask questions was a way of seeking clarification on information provided by clinicians and of opening up a dialogue, where patients could also offer information about themselves and their concerns.

The subtext of the two-way exchange during consultations was to build mutual understanding and a commitment to work in partnership during the protracted treatment process. As Sandman and Munthe suggest, ‘decision-making should be shared in order to expand the possibilities for patient autonomy without abandoning the patient or giving up the possibility to influence how the patient is benefitted’ (p.290) [2]. Having an established space for communication allowed clinicians and patients to work out and negotiate decisions in such a way that patients’ and clinicians’ values became better aligned.

For example, clinicians who felt that patients’ preferences were not in their best interest were able to establish a way to meet patients’ fundamental values in decision-making. *Ingrid,* diagnosed with a benign tumour, learnt about a cosmetic breast reconstruction practice that made a surgical incision from the armpit, from a reality television programme about plastic surgery, which she understood was beneficial in reducing unsightly scaring. The aesthetic preservation of *Ingrid’s* breasts was important for her because: ‘I don’t want to have any deformed part of me … when it comes to your breasts – it’s my favourite part of myself. So I don’t want it to be ugly.’ However, her clinician advised against this type of surgical option, as he felt it was not appropriate for her and for the position of her benign tumour, and told her that it would damage healthy tissues unnecessarily. The clinician explained to *Ingrid* how the surgical option did not suit her situation, and that the appearance of her breasts would be minimally altered by his planned surgery that was isolated to the nipple-areola region. *Ingrid* accepted this advice over time, and settled with the original surgical plan, as she felt reassured that the operation would cause minimal scarring. This was an explanation that made *Ingrid* ‘feel better’ about the decision, which she eventually described as her ‘own choice’.

Through the opportunity for dialogue and exchange of information about her values and preferences with her clinician, *Ingrid* developed a better understanding of the clinician’s reason for suggesting a more appropriate type of surgery that would achieve a clinical outcome as well as a cosmetic one. This communication process facilitated *Ingrid*’s acceptance of a change to her plans and led to her feeling a sense of ownership over the final treatment decision, even though the final decision about the type of surgery differed to the one she originally envisaged.

### Lack of clarifying values

There were exceptions to the negotiated and shared understanding described above, and times when a lack of clarification of patients’ personal values about treatment decisions in their relationship with family members and clinicians did result in a less than ideal decision-making scenario.

*Annette* was deeply challenged by the conflicting preferences of her adult daughter and her clinician about the best type of surgical option. Initially, she was happy to follow her clinician’s advice to undergo several courses of chemotherapy to reduce the size of her tumour in preparation for lumpectomy. This would have allowed her to preserve most of her breast, instead of the more drastic surgical option of mastectomy, which her clinician felt was unnecessary given the location and relatively small area of tumour invasion. However, during chemotherapy, *Annette’s* distressed adult daughter asked her to change her mind and undergo mastectomy, to ensure a higher chance of survival, after seeing a documentary about a high-profile American celebrity who had undergone mastectomy to treat breast cancer. Having had an emotional conversation with her daughter, *Annette* asked her clinician about this procedure, to be told that the risk of cancer recurrence would be the same for both types of surgery. While *Annette* settled with the clinician’s advice, she began to doubt the reliability of the information she was given by her clinician. Pulled in several directions by her intersecting relationships with her daughter and her clinician, she remained unsure of what her own personal preference was. Without enough self-confidence, she was uncomfortable with the decision to continue with chemotherapy and lumpectomy, even though she remained compliant.

### Gaining experience and self-reflection over-time

As patients progressed with breast disease care, they developed more understanding and experience of medical treatment. Gradual progress towards achieving treatment goals also added to a sense of self-confidence that departed from the position of a novice and feelings of vulnerability, which were more common at the beginning of treatment. As *Caitlyn* explained: ‘Once treatment started, I started to feel better psychologically, because you feel like you’re taking control and you’re fighting back.’

Over time, patients developed better awareness of their own preferences and values in care options. According to *Ella,* she had become more assertive in her current treatment, having realised that her past experience of not questioning what turned out to be a misdiagnosis of another disease by a previous doctor had not helped her case. After her cancer diagnosis, she became worried about the risk of having bone cancer linked to breast cancer, and the chance of becoming pregnant during chemotherapy. *Ella* requested additional assessments to rule out bone cancer, and requested a referral to have her ovaries removed. While her oncologist suggested that neither procedure was necessary, as *Ella*’s risk of bone cancer was low and the removal of ovaries was drastic and permanent, he nevertheless supported her decision, given her insistence and her desire not to have any more children. *Ella* felt that the support demonstrated by her clinician gave her the ‘peace of mind’ to continue with breast cancer treatment.

At 72, *Hillary* was the oldest patient in the study, and was undergoing treatment for a second cancer in her left breast, just months after she concluded chemotherapy, lumpectomy and radiation treatment for cancer in her right breast. She felt her previous negative experience of chemotherapy affected her decision to opt for a mastectomy rather than chemotherapy during this second treatment. At the beginning of her treatment of the right breast, *Hillary* adhered to the advice of undergoing chemotherapy. However, its side-effects turned out to be debilitating. As a result, she was determined not to receive chemotherapy again, even though it was offered, should she want to preserve the left breast. Instead, she opted for a mastectomy as she ‘just wanted to get rid of the whole thing’, rather than to have to endure the side-effects of chemotherapy and 5 weeks of intensive and tiring radiation treatment at her age.

Personal experience incrementally gained from treatment helped patients gain clarity about their preferences and values in terms of ongoing treatment choices. This facilitated a sense of better control over the path of their treatment and helped patients develop a stronger sense of self-trust over time. However, this required clinicians, as key players in treatment, to enable a communicative space for patients to discuss and negotiate treatment choices that may divert from standard medical advice, and allow patients to feel at ease that their choices are supported by medical professionals.

The study results also highlighted an interesting phenomenon. The experience of confronting illness and undergoing treatment can give rise to a transformative shift in building assertiveness and self-trust in response. Previously, *Ella* had adopted the socially accepted norm of the patient who complied with clinicians’ directives. However, in light of her negative experience of misdiagnosis, her attitude changed: ‘I’ve learned to push for what I want now!’ Similarly, the experience of cancer diagnosis gave *Caitlyn* the realisation that as a mother over-burdened by her role as the primary carer, she had neglected her own health in the interest of looking after her young family:

*Being a mother of three girls, I was putting myself last. Now I realise that was so stupid … in order to look after my family I need to put myself first. I feel like this is a huge wakeup call and almost a bit of a gift in that if this hadn't happened, I could have merrily gone along … You never know.*

## Discussion

**Fig. 1:**
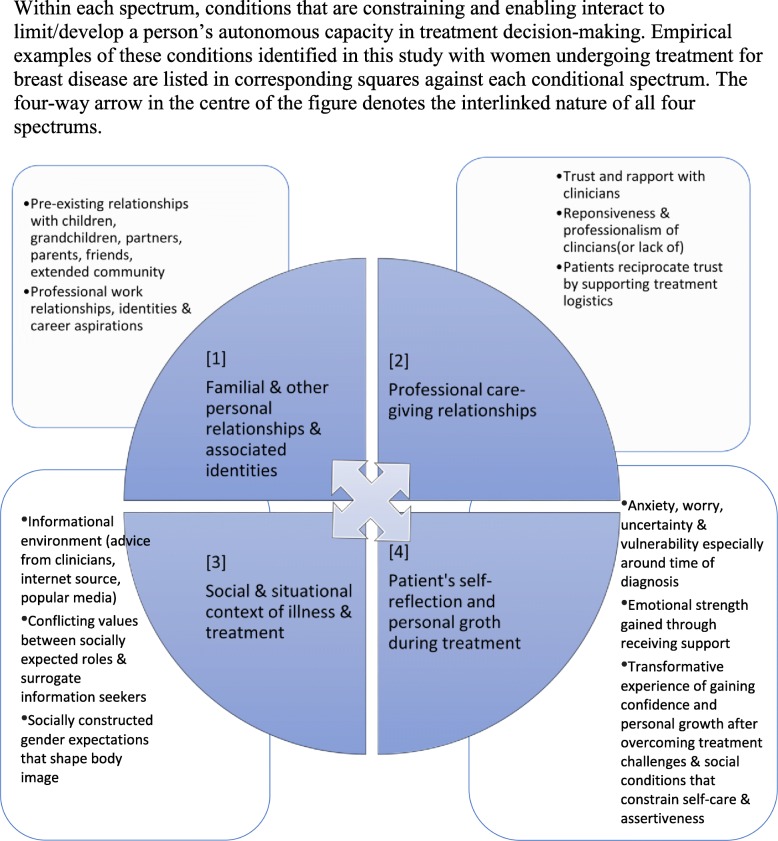
Four conditional spectrums of relational autonomy

Relational autonomy emphasises the social conditions and relationships that underscore a person’s self-identity and capacity for decision-making. The decisional environment of a patient should be understood beyond isolated events in the clinic, but as a part of a wider narrative of illness, shaped by personal history and experience situated in a dynamic social world [26]. This study illuminated the dynamic interplay between the constraining and enabling social conditions that contextualise the formation of personal values and relational interactions that lead to treatment decision-making. These social conditions that limit and facilitate capacity for autonomy are better conceptualised as a spectrum of fluid interactions between constraining and enabling elements of autonomy. In view of this, and arising from our results, four interlinked elements that constitute a person’s relational environment are presented in Fig. 1. These are (1) family and other personal relationships and identities; (2) professional care-giving relationships; (3) the social and situational context of illness and treatment; and (4) patients’ self-reflection and personal growth during treatment. The empirical examples of these conditions, as examined in this study, are outlined in corresponding boxes against each of the spectrums.

The socially produced and largely gendered relational identities of, for example, being a mother, partner and career professional underscore women’s desire to undergo treatment for breast disease (Spectrums 1 and 3). In turn, the social and relational resources offered by “important social others” [12] such as family members, friends and employers were crucial sources of support. Patients’ need to rely on others in assisting with decision-making arose from the self-recognition that their capacity for rational decision-making is greatly diminished at a time of trauma and vulnerability [10]. However, this is not because of a lack of capacity for deliberative reasoning on the part of the patient, but the active and reasoned use of relational resources by patients, drawing on the rational capacity of others in whom they trust. This is in order to achieve treatment goals that are beneficial to not just patients, but also their loved ones, who are immediately implicated by the patient’s health and wellbeing.

Clinicians occupy a socially privileged position of power in relation to lay patients [7, 25], to whom they look for medical expertise for guidance (Spectrums 2 and 4). However, patients demonstrated considerable discernment in response to the way which clinicians attempt to build trust and establish interpersonal rapport. It is the communicative skill of clinicians to open a space for mutual exchange of information and values that is most crucial. This promotes the ongoing collaborative approach to address and negotiate treatment decisions and challenges as they arise. As Joseph-Williams, Elwyn and Edwards [27] suggest, the knowledge gained from information provision in routine clinical consultations does not necessarily help patients to participate more fully in decision-making. Rather, it is the opportunity and encouragement to become more active and engaged in the decision-making process that helps patients gain a sense of self-growth and autonomous capacity. In view of this, patient-clinician communication should be understood beyond the transaction of information that simply supports patients’ informed decision-making, to a process of strengthening the relational conditions for trust-building and negotiation of shared values [11].

Socially produced gender norms that shape women’s attitudes about body image and healthcare are reflected in popular media and various internet-based information sources. These conditions in the informational environment can influence the values and preferences of both patients and close family on treatment decisions [26]. Family members and friends can play the role of ‘surrogate seekers of information’ on behalf of patients (Spectrum 1 and 3) [28], but in turn can create undue pressures and decisional conflicts for patients. These pressures culminated in the case of *Annette,* whose desire to engage with the preferences of her daughter and her doctor came into conflict (Spectrums 1, 2, 3 and 4).

Given their position of power and influence, clinicians can help patients clarify the values and emotions underpinning their preference for treatment, as well as the values and beliefs of supportive others. In many cases, treatment options not envisaged by patients may still enable patients to feel they have achieved their goals. Rather than discourage patients from accessing internet-based information, in many cases clinicians can support patients’ ongoing information-seeking needs by guiding them in appropriate information-selection that is suitable to their own personal and medical circumstances [29]. Additional evidence-based decisional resources are now increasingly available for achieving these objectives. For example, the Breast RECONstruction Decision Aid (BRECONDA) is an interactive decision tool designed in Australia shown to assist in clarifying patients’ values and preferences, and reducing patient distress associated with decisional conflicts when multiple options of treatment are available [17, 30].

Finally, a relational approach to autonomy highlights the feminist concern that women’s decision-making can be adversely influenced by constraining social norms and power relationships [6, 7, 10, 31]. In this study, these include the pressure to conform to gendered expectations of body image, the over-burden of the primary carer of children, and the lack of confidence to question the authority and knowledge of clinicians who hold a position of power. In light of the social injustices revealed by a relational approach to autonomy, Sherwin [7] urges the need to transform the “status quo” of structural patterns of social condition *and* personal notions of selfhood that currently constrain the autonomous capacity of women. This study shows that women can develop a capacity to recognise and question the socially conditioned limitations they experience during the course of living with illness and undergoing treatment. In the case of *Ella* and *Caitlyn*, for example, it is through reflecting on the experience of adversity that gave rise to a self-realisation to prioritise their own needs over those of others. This is both a personal process of self-reflection (Spectrum 4), and a social process of responding to opportunities or challenges in the social structure within which a person exists (Spectrum 3) [2, 12, 32]. Indeed, the social transformation that challenges the status quo of gender norms and power relations is taking place at the personal level, and in this way, becomes meaningful in women’s everyday lives.

## Conclusion

This study applies the concept of relational autonomy to empirical qualitative data drawn from the setting of routine clinical consultations around breast disease treatment. It supports a person-centred approach to care, by illustrating how patients’ experiences of treatment are contextualised by personal relationships and life stories. Furthermore, their encounters of illness and treatment in the clinic made visible both experiences of distress and sources of strength that continues to transform their lives.

The results of the study developed a conceptual model of four conditional spectrums of relational autonomy, which can be transferred and applied to a range of other social and situational contexts. The constraining and enabling conditions of relational autonomy can also be identified in different forms, with different patient cohorts and types of treatment decisions. This is particularly salient, given that relational autonomy is characterised by its sensitivity to social contexts and conditions.

While this study is limited by being relatively modest in scale, and set in a specific, socio-cultural location in Sydney, Australia with English-speaking female patients who are 18 years or older, its strengths lie in its credibility, and the contextual illustration of relational autonomy. The findings nevertheless inform future, larger scale studies that will include culturally and linguistically diverse populations, and patients who may vary more in age and financial status, to understand wider factors that influence relational autonomy.

## Additional file

Additional file 1: Patient interview guide, a list of interview topics used to direct discussions with patients during interviews. (DOCX 13 kb)

## Abbreviations

BRECONDA: Breast RECONstruction Decision Aid
DCIS: Ductal carcinoma in situ

## References

[1] Hogden A (2014). Optimizing patient autonomy in amyotrophic lateral sclerosis: inclusive decision-making in multidisciplinary care. Neurodegener Dis Manag.

[2] Sandman L, Munthe C (2009). Shared decision-making and patient autonomy. Theor Med Bioeth.

[3] Barry MJ, Edgman-Levitan S (2012). Shared decision making—the pinnacle of patient-centered care. N Engl J Med.

[4] Falkum E, Forde R (2001). Paternalism, patient autonomy, and moral deliberation in the physician-patient relationship: attitudes among Norwegian physicians. Soc Sci Med.

[5] Blackler L (2016). Compromised autonomy: when families pressure patients to change their wishes. J Hosp Palliat Nurs.

[6] Mackenzie C, Stoljar N. Relational autonomy: feminist perspectives on autonomy, agency, and the social self. New York: Oxford University Press; 2000.

[7] Sherwin S, Sherwin S (1998). A relational approach to autonomy in health care. The politics of women's health: exploring agency and autonomy.

[8] Walter JK, Ross LF (2014). Relational autonomy: moving beyond the limits of isolated individualism. Pediatrics.

[9] Entwistle VA, Carter SM, Cribb A, McCaffery K (2010). Supporting patient autonomy: the importance of clinician-patient relationships. J Gen Intern Med.

[10] McLeod C, Sherwin S, Mackenzie C, Stoljar N (2000). Relational autonomy, self-trust, and health care for patients who are oppressed. Relational autonomy: feminist perspectives on autonomy, agency, and the social self.

[11] Entwistle VA, Watt IS (2013). Treating patients as persons: a capabilities approach to support delivery of person-centered care. Am J Bioeth.

[12] Ruhe Katharina M., De Clercq Eva, Wangmo Tenzin, Elger Bernice S. (2016). Relational Capacity: Broadening the Notion of Decision-Making Capacity in Paediatric Healthcare. Journal of Bioethical Inquiry.

[13] Ho A (2008). Relational autonomy or undue pressure? family’s role in medical decision-making. Scand J Caring Sci.

[14] Bell JA, Balneaves LG (2015). Cancer patient decision making related to clinical trial participation: an integrative review with implications for patients’ relational autonomy. Support Care Cancer.

[15] Meadow SL (2015). Defining the doula’s role: fostering relational autonomy. Health Expect.

[16] Reyna VF, Nelson WL, Han PK, Pignone MP (2015). Decision making and cancer. Am Psychol.

[17] Sherman KA, Shaw L-KE, Winch CJ, Harcourt D, Boyages J, Cameron LD, Brown P, Lam T, Elder E, French J (2016). Reducing decisional conflict and enhancing satisfaction with information among women considering breast reconstruction following mastectomy: results from the BRECONDA randomized controlled trial. Plast Reconstr Surg.

[18] Rapport F, Khanom A, Doel M, Hutchings HA, Bierbaum M, Hogden A, Shih P, Braithwaite J, Clement C (2018). Women’s perceptions of journeying toward an unknown future with breast cancer: the “lives at risk study”. Qual Health Res.

[19] Twomey M (2011). Relational autonomy: an example from breast cancer nursing. Ethics Soc Welf.

[20] Siminoff LA, Step MM (2005). A communication model of shared decision making: accounting for cancer treatment decisions. Health Psychol.

[21] Hesse-Biber S (2014). The genetic testing experience of BRCA-positive women: deciding between surveillance and surgery. Qual Health Res.

[22] Martinez KA, Kurian AW, Hawley ST, Jagsi R (2015). How can we best respect patient autonomy in breast cancer treatment decisions?. Breast Cancer Manag.

[23] Rapport F, Shih P, Bierbaum M, Hogden A. Schema analysis: a teamwork approach. In: Liamputtong P, editor. Handbook of research methods in health social sciences. Singapore: Springer; 2018.

[24] Rapport F (2010). Summative analysis: a qualitative method for social science and health research. Int J Qual Methods.

[25] Shih P, Worth H, Travaglia J, Kelly-Hanku A (2017). Pastoral power in HIV prevention: converging rationalities of care in Christian and medical practices in Papua New Guinea. Soc Sci Med.

[26] Clayman ML, Gulbrandsen P, Morris MA (2017). A patient in the clinic; a person in the world: why shared decision making needs to center on the person rather than the medical encounter. Patient Educ Couns.

[27] Joseph-Williams N, Elwyn G, Edwards A (2014). Knowledge is not power for patients: a systematic review and thematic synthesis of patient-reported barriers and facilitators to shared decision making. Patient Educ Couns.

[28] Cutrona SL, Mazor KM, Vieux SN, Luger TM, Volkman JE, Rutten LJF (2015). Health information-seeking on behalf of others: characteristics of “surrogate seekers”. J Cancer Educ.

[29] Coulter A, Oldham J (2016). Person-centred care: what is it and how do we get there?. Future Hosp J.

[30] Sherman KA, Harcourt D, Lam T, Shaw L, Boyages J (2014). BRECONDA: development and acceptability of an interactive decisional support tool for women considering breast reconstruction. Psycho-Oncology.

[31] Shih P, Worth H, Travaglia J, Kelly-Hanku A (2017). ‘Good culture, bad culture’: polygyny, cultural change and structural drivers of HIV in Papua New Guinea. Cult Health Sex.

[32] Mackenzie C (2008). Relational autonomy, normative authority and perfectionism. J Soc Philos.

