# Supplementary material for: Relational autonomy in breast diseases care: a qualitative study of contextual and social conditions of patients’ capacity for decision-making
Source: BMC Health Serv Res. 2018 Oct 25;18:818. doi: 10.1186/s12913-018-3622-8 (PMC6202865; doi:10.1186/s12913-018-3622-8)
Supplement: Supplementary file 1 — Patient interview guide, a list of interview topics used to direct discussions with patients during interviews. (DOCX 13 kb) [file 12913_2018_3622_MOESM1_ESM.docx]

Patient interview guide

1. Experiences and views of receiving information about treatment and risk in oncology consultation
2. How often and which healthcare professionals consulted since treatment
3. Changes of views and sense of clarity about treatment since early stages of diagnosis
4. Dealing with uncertainty around risk in daily life during treatment; How this may have changed since first diagnosis and during treatment
5. Impact of concerns regarding risk on patients and family members/friends
6. Sources of information about treatment and risk
7. Treatment decisions, how they are made and by whom, how care is negotiated with healthcare professionals
8. Views on the way in which consultation and treatment pathways have proceeded so far
9. Aspirations for future health and well-being / health and wellbeing of other family members.
